# Development and optimization of NIRS prediction models for simultaneous multi-trait assessment in diverse cowpea germplasm

**DOI:** 10.3389/fnut.2022.1001551

**Published:** 2022-09-23

**Authors:** Siddhant Ranjan Padhi, Racheal John, Arti Bartwal, Kuldeep Tripathi, Kavita Gupta, Dhammaprakash Pandhari Wankhede, Gyan Prakash Mishra, Sanjeev Kumar, Jai Chand Rana, Amritbir Riar, Rakesh Bhardwaj

**Affiliations:** ^1^Division of Plant Genetic Resources, ICAR-Indian Agricultural Research Institute, New Delhi, India; ^2^Division of Germplasm Evaluation, ICAR-National Bureau of Plant Genetic Resources, New Delhi, India; ^3^Division of Plant Quarantine, ICAR-National Bureau of Plant Genetic Resources, New Delhi, India; ^4^Division of Genomic Resources, ICAR-National Bureau of Plant Genetic Resources, New Delhi, India; ^5^Division of Genetics, ICAR-Indian Agricultural Research Institute, New Delhi, India; ^6^Division of Bioinformatics, ICAR-Indian Agricultural Statistics Research Institute, New Delhi, India; ^7^Alliance of Bioversity International and CIAT, Region-Asia, India Office, New Delhi, India; ^8^Department of International Cooperation, Research Institute of Organic Agriculture FiBL, Frick, Switzerland

**Keywords:** MPLS regression, germplasm screening, nutritional composition, RPD, RSQ_external_

## Abstract

Cowpea (*Vigna unguiculata* (L.) Walp.) is one such legume that can facilitate achieving sustainable nutrition and climate change goals. Assessing nutritional traits conventionally can be laborious and time-consuming. NIRS is a technique used to rapidly determine biochemical parameters for large germplasm. NIRS prediction models were developed to assess protein, starch, TDF, phenols, and phytic acid based on MPLS regression. Higher RSQ_external_ values such as 0.903, 0.997, 0.901, 0.706, and 0.955 were obtained for protein, starch, TDF, phenols, and phytic acid respectively. Models for all the traits displayed RPD values of >2.5 except phenols and low SEP indicating the excellent prediction of models. For all the traits worked, *p*-value ≥ 0.05 implied the accuracy and reliability score >0.8 (except phenol) ensured the applicability of the models. These prediction models will facilitate high throughput screening of large cowpea germplasm in a non-destructive way and the selection of desirable chemotypes in any genetic background with huge application in cowpea crop improvement programs across the world.

## Introduction

Legumes have high nutritional qualities, are suitable for soil health and show resilience to climate change; these attributes can help to attain food security among low income developing nations of the world. Cowpea (*Vigna unguiculata* (L.) Walp.) is one such multipurpose legume originating from Africa (1), that may facilitate in providing food security and is adaptable to climate change and harsh conditions (2), becoming a successful crop in arid and semi-arid areas. In Sub-Saharan Africa, Asia, and parts of America, it is a significant pulse crop, grossing a total world production of 8.9 million metric tons (3). Nigeria accounts for 40% of total cowpea production, followed by Niger (26.8%) and Burkina Faso (7.3%). In India, it is grown as a minor pulse in an area of 3.9 mha with a production of 2.21 million tons (4), mainly in the arid and semi-arid tracts of Haryana, Punjab, Delhi, and Western Uttar Pradesh.

Owing to its high nutritional value, cowpea can be a good food source to combat malnutrition in low income developing countries, especially in Asian and African countries (5). It is rich in protein (24/100 g), total dietary fiber (11/100 g), carbohydrates (60/100 g), and low in fatty acids (<2/100 g), with a significant amount of essential amino acids^1^. High starch in cowpea can be used to make processed products like moin-moin and akara (6). It is a leguminous crop rich in TDF (16–20 /100 g), lowering the risk of cardiovascular diseases, diabetes and heart ailments (7). A number of bio-functional non-nutrients are present in dry cowpea seeds like phytates, flavonoids, and tannins (8). Polyphenols are present in an abundant proportion in legumes, which helps in imparting anti-oxidant properties ranging from 46.5 to 119.6 mg GAE/100 g (9, 10). Phytates are distributed widely in cereals and legumes, mostly stored in the form of phosphate in seeds. It has the ability to chelate divalent cations like Fe, Mg, and Cu, decreasing the bioavailability of the minerals. In cowpeas 0.5–3/100 g phytates has been reported (8, 11).

Huge variability exists in the nutritional attributes of cowpea, i.e., starch, protein, phenolics, phytates, TDF, and even in micronutrients (12); along with genetic relationships the variability in biochemical traits could help to develop new cultivars with superior traits. Despite the fact that crop improvement programs produce a large number no. of crosses/lines each year, but it is difficult to evaluate conventionally through complex methods which are labor intensive, time taking and technically complex. For the evaluation of a large number of accessions, NIRS has been proven to be a better technique. NIRS is a non-destructive technique widely used to predict organic compounds of grain material based on electromagnetic radiation (13). It is known for various advantages when compared to traditional procedures, including rapid determination, non-destructive, minimal usage of reagents, and less analysis of costs (14). Large scale rapid screening previously has been done for fatty acid profile in groundnut, major wood characteristics in Eucalyptus (15, 16). Electromagnetic radiation from 780 to 2,500 nm in the NIR region is covered by this technique (17), and absorption of IR light by the substance under examination is the basis for infrared spectroscopy. Molecular vibrations and rotation are caused by absorption and relative proportion of C–H, N–H, O–H, which forms the primary structure of biomolecules with a frequency similar to those found in the infra-red region of the electromagnetic spectrum (18, 19). These bands are important for identifying molecular interaction between functional groups and obtaining chemical information about the organic substance (20). Establishing a spectrochemical prediction known as spectrum calibration, multivariate regression methods, also known as chemometrics, are utilized to calibrate the NIR spectra to these organic elements due to the diversity of organic compounds extensively in biomaterials (21). The biochemical information included in a substance spectrum characteristic is separated from the physical or chemical information revealed by reference lab values via calibration (22). The analytical capacity of NIRS was determined by the relation between the number of biochemical parameters and the corresponding absorption spectra. Authentication and validation of NIRS prediction models depend on the accuracy of the relationship through pre-processing the spectral data and multivariate statistical analysis. Multiplicative scatter correction (MSC), standard normal variate and detrend (SNV–DT) are common pre-treatment steps (23), whereas multivariate regression techniques like partial least square (PLS), modified PLS (MPLS) and principal component regression (PCR) characterize the relation between biochemical components and spectral data (22).

Among legumes, NIRS prediction models have been developed for physicochemical properties, fatty acid and mineral composition in lentil (24, 25), phytates in green gram (18), neutral detergent fiber and acid detergent fiber fraction in chickpea (26), nutritional quality for chickpea straw (27). In cowpea, NIRS models have been developed to predict nitrogen content in cowpea seed (12, 28), and crude protein content in cowpea leaves (14).

In this study a combination of pre-processing methods (derivatives, gaps, and smoothening) along with MPLS regression have been used with an objective of developing robust NIRS prediction models for five biochemical traits, i.e., protein, starch, TDF, phenols, and phytates, to access the nutritional diversity in cowpea germplasm. These models could be applicable in different sectors of food industry, high throughput screening in national and international gene banks, seed industries and facilitate breeders in crop improvement programs.

## Materials and methods

### Sample collection

Four hundred seventy-five accessions of cowpea consisting of indigenous and exotic collections were taken from MTS of National Gene Bank at ICAR-NBPGR, New Delhi, India, having high variability in seed morphology. Using Augmented Block Design accessions were grown in Issapur experimental farm, New Delhi, India, following standard agronomic practices (29). Matured and dried seeds were collected, and extraneous material was removed.

### Sample selection for NIRS modeling

Using FOSS NIRS 6500, 475 accessions were scanned, and the reflectance spectrum was recorded from 400 to 2,400 nm. Hierarchical clustering was done by Ward's method using squared Euclidean distance of 5 on the normalized spectral data of 475 samples. Major clusters and sub-clusters were identified and separated in the same manner. A set of highly diverse 121 accessions were selected to cover the entire range of variability in the data set. The wet chemistry values of these accessions were used as reference values. These samples were homogenized, ground, and sieved through a 1 mm sieve in FOSS cyclotec, and the flour thus obtained was used for scanning and wet chemistry analysis.

### Generation of reference data for NIRS prediction models

#### Total protein content

Kjeldahl method (AOAC 984.13) (30) was used to estimate total nitrogen content where FOSS Tecator 2300 Kjeltec Analyser Distiller Unit) was used. %N was converted to percent protein using Jone's conversion factor of 6.25.

#### Total starch content

Total starch content was estimated by Megazyme total starch assay kit as per AOAC 996.11 (30) which uses α-amylase, amyloglucosidase and glucose oxidase peroxidase. Absorbance was recorded at 510 nm using a UV-VIS spectrophotometer, and the results were expressed in g/100 g.

#### Total dietary fiber (TDF)

TDF was estimated using a commercial assay kit from Megazyme International, Wicklow, Ireland (AOAC 985.29) (30), which includes the use of α-amylase, amyloglucosidase, and protease followed by precipitation with 95% ethanol and the results were expressed in g/100 g.

#### Total phenolics

Total phenolics was estimated by Folin Ciocalteau Reagent assay (31), which includes both oxidation and reduction reaction. Absorbance was recorded at 650 nm using a UV-VIS spectrophotometer, and the results were expressed in GAE g/100 g.

#### Phytates

Phytates was estimated using commercial assay kit from Megazyme International, Wicklow, Ireland (AOAC 986.11) (30) with the use of phytase and alkaline phosphatase. Absorbance was recorded at 655 nm, and the results were expressed in g/100 g.

#### Quality control

All the estimations were carried out in duplicates to ensure replicability and accuracy of the results. To ensure accuracy suitable standards and reagents blanks were used for each biochemical parameter. ASFRM-Rice-2 from PT-8 (INMU, Thailand) was used to validate protein and TDF. Total starch control kit (K-TSCK) control flours (wheat, maize starch) were used for validation of total starch. Total phytic acid kit control oat flour was used as standard reference material for phytic acid.

### Spectroscopic analysis

FOSS NIRS 6500 spectrophotometer equipped with Win ISI Project Manager Software Version 1.50 was calibrated using a reference tile (100% white). Approximately 5 gm homogenized samples were loaded and scanned in a circular ring cup with a quartz window (3.8 cm and 1 mm thickness). The average spectrum was recorded by scanning the sample 32 times at 400–2,500 nm and was registered as log (1/R) at increments of 2 nm, where *R* is the respective reflectance.

### Development of calibration and validation sets

For development of calibration and validation set, the accessions were arranged in ascending order and every second value was taken out to make the calibration set. Therefore, calibration and validation sets were obtained in the ratio of 2:1, which ensured uniform variability in both the sets (32). Thus, the accessions were divided into two sets for modeling, i.e., 81 accessions in the training set and 40 accessions in the validation set for all the traits.

### Calibration and validation of equations

Win ISI Project Manager Software Version 1.50 was used to develop calibration equations using multivariate analysis by regressing spectral data with laboratory values. MPLS regression with cross-validation was used to develop equations on the above software using full spectra. Various mathematical algorithms, such as SNV–DT (SNV with detrend) were used for scatter correction and pre-processing the spectral data for each biochemical parameter. Moreover, different mathematical treatments like “2,4,6,1”, “2,8,8,1”, “2,4,4,1”, “3,4,4,1”, and “2,8,8,1” were used to develop models where the first digit represents the order of derivative; the second digit represents the gap (data points), third and fourth digits represents the data point in first and second smoothening. The developed calibration equations were assessed by different parameters such as coefficient of determination (RSQ), standard error of cross-validation [SEC(V)], standard deviation (SD), one minus variance ratio (1-VR). SEC(V) of RSQ_internal_ was calculated by Win ISI Project Manager Software V 1.50. The portion of the variation in reference data that may be characterized by the variance in predicted data is displayed using RSQ. High RSQ and lower SEC models are superior to low RSQ and higher SEC values. Only cross-validation was insufficient to assess the accuracy of the models [i.e., with SEC(V) and 1-VR], so RSQ_external_ (coefficient of determination in external validation), bias (difference between predicted and reference values), SEP (standard error of performance), SEP(C) (corrected standard error of performance) and RPD (ratio of performance to deviation) values are used. RPD values are used for accuracy of MPLS models where if RPD <1.5, the model is not reliable, in between 1.5 and 2.0, it indicates the capacity of a model to distinguish high and low values, in between 2 and 2.5, indicating approximate quantitative prediction, in between 2.5 and 3.0, indicates good quality prediction and if it is >3.0, then the prediction is excellent (13).

### Statistical analysis

All of the calibration and prediction was done using Win ISI III Project Manager Software Version 1.50, which applied various mathematical treatments based on spectral and analytical data. Reference and predicted values were monitored using Win ISI Project Manager Software V 1.50 with the developed equation. Using global statistical values like RSQ, slope, bias, RPD and SEP(C), the accuracy and predictive capacity of the model were evaluated. The coefficient of determination (RSQ_internal/external_) was externally plotted using Veusz statistical package for graphs of all the biochemical parameters.

A paired sample *t*-test was performed between reference and predicted values at 95% confidence interval using Jamovi statistical software package v1.6.9 (33). Strict parallel analysis was performed to check the reliability of the developed models and the reliability score was calculated by IBM SPSS v17.3 between the predicted and laboratory validated samples.

## Results and discussion

### Quantification of biochemical parameters

**Figure 1:**
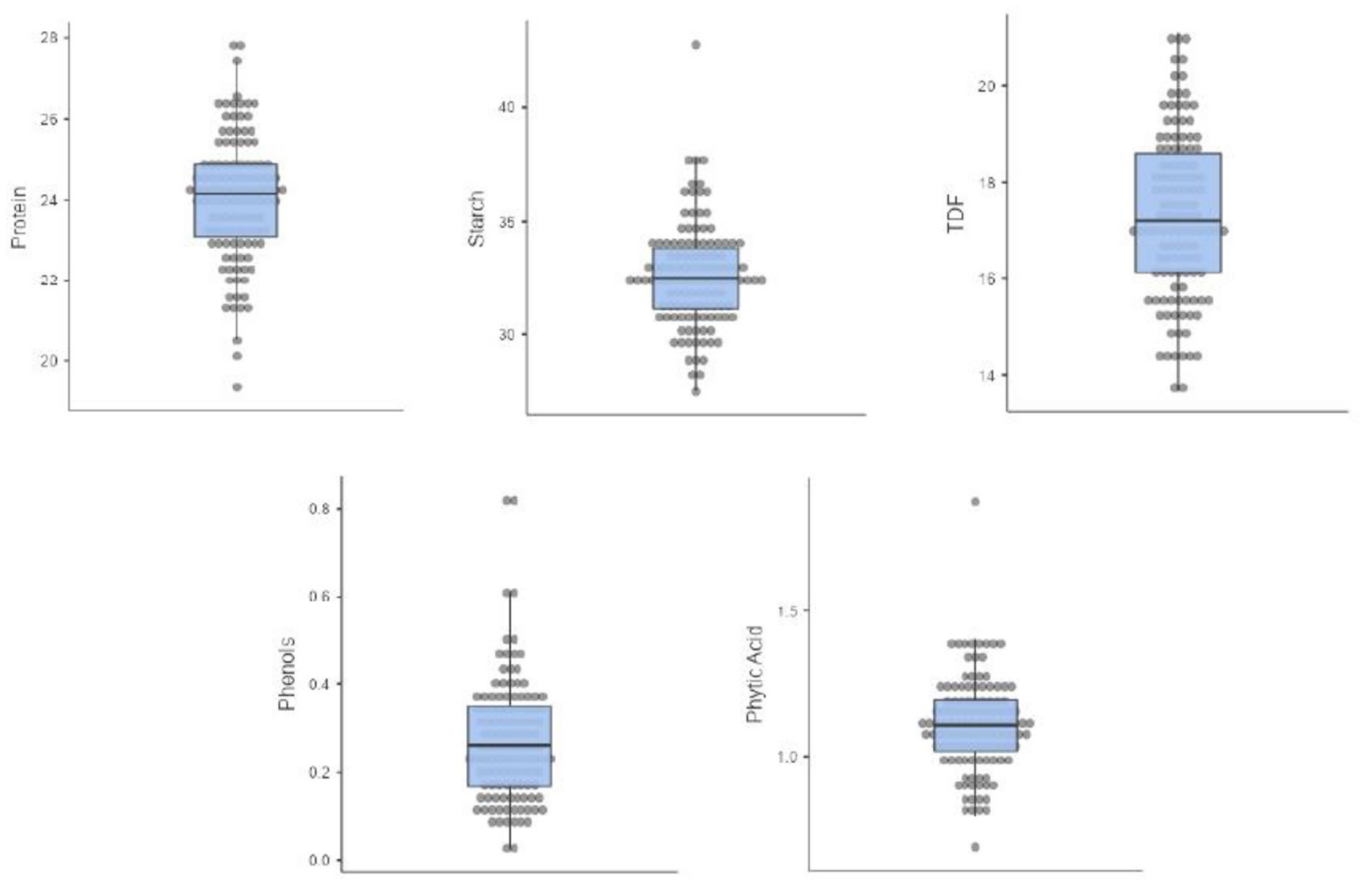
Box and whisker plots of 121 cowpea germplasm showing the distribution of protein, starch, TDF, phenols, and phytic acid.

Five nutritional traits were worked out for 121 diverse cowpea accessions, and descriptive statistics are given in Table 1, whereas the box and whisker plots to showcase the variability of each trait are given in Figure 1. Protein was found to be range from 20 to 27.8/100 g, which is the most important trait for any legume. It is within the interval previously reported from 17.4 to 31.7/100 g (34) but lower than the values of 28.1–31.8% for five different Ethiopian cultivars (35). The protein content of cowpea has essential amino acids like lysine, histidine, and aromatic amino acids (36, 37). Starch content varied from 26.7 to 38.7/100 g, which is in agreement with the range of 28.3–36.2/100 g reported (34) but is lower than compared to other legumes like black gram 45/100 g and red bean 46 /100 g (38). Starch content will be useful for making processed products like moin-moin and akara. TDF in our study significantly varied from 12.2 to 22.4/100 g with a mean of 17.3 /100 g, close to the reported values worked by Akissoe et al. (15.6/100 g) but lower than worked values of 27.4/100 g (39, 40). Higher TDF in legumes corresponds to the lowering of cardiovascular diseases, diabetes, obesity, etc. Anti-nutritional factors such as phenolic compounds like phenolic acids, tannins, flavonoids, and phytates were analyzed and found in the range of 0.08–0.545 GAE/100 and 0.583–1.62/100 g, respectively. These compounds, if present in higher quantities, can limit the bioavailability of divalent cations (41).

**Table 1:** Descriptive statistics of total protein, starch, TDF, phenols, and phytic acid.

	**Total dietary fiber%**	**Starch%**	**Protein%**	**Phytate%**	**Phenols%**
*N*	121	121	121	121	121
Mean	17.3	32.6	24.0	1.11	0.272
Standard deviation	1.71	2.27	1.54	0.163	0.139
Minimum	13.7	27.5	19.4	0.690	0.03
Maximum	21.1	42.7	27.9	1.88	0.832

### NIRS spectra acquisition

**Figure 2:**
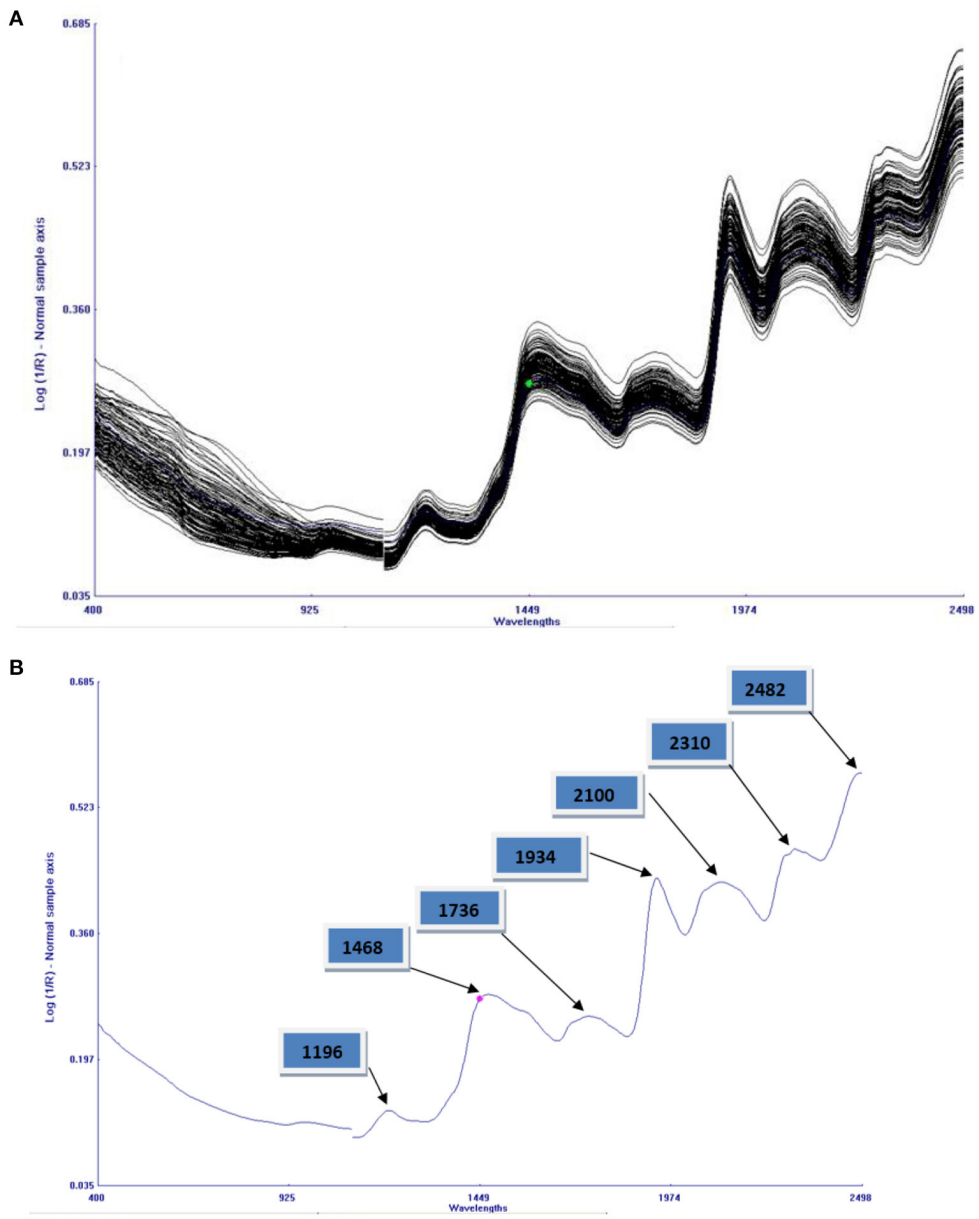
**(A)** A combined plot of reflectance spectra of all the entire 121 cowpea germplasm. **(B)** An average reflectance spectrum of cowpea homogenized flour with seven absorption bands.

Combined NIRS spectra of 121 cowpea accessions in the range of 400–2,490 nm is given in Figure 2A. The bands result from overlapping absorption that corresponds to the combination and overtones of vibrational modes N–H, O–H, and C–H, found in proteins, fatty acids, and carbohydrates, respectively. The main absorption bands were observed at 1,196, 1,468, 1,736, 1,934, 2,100, 2,310, and 2,482 nm, as shown in Figure 2B. C–O and N–H stretch, found in the spectral region between 2,000 and 2,222 nm, denoting protein content (42). O–H group can also be found in 1,560–1,640 nm, which can be allocated to the O–H group associated with phytates (18). O–H stretch first overtone of hydroxyl phenol groups present in 1,430–1,470 nm, whereas O–H bending/stretching of polysaccharides was found near the peak of 1,920 nm. The third polysaccharide overtone stretched by asymmetric C–O–O stretch was found near 2,083 nm (43). Unclear peaks were observed near 2,304–2,352 nm; this wavelength characterizes fatty acids and oils; since cowpea has little number of fatty acids, the bands were unclear. Symmetric stretching (–CH) in methyl groups (–CH_3_) found in a wavelength of 1,200 nm causing weak absorption bands. Similar bands were found in the study of rice flour and its quality properties using NIRS (44).

### Calibration of NIRS model

A calibration set is generally referred to as training set providing learning and training to build the model. Regression algorithms like MPLS, PLS, and PCR can be used for model development, but as compared to the PLS algorithm, MPLS is supposed to be more stable and accurate (24) and thus was employed in the present study. Both spectra and reference composition were used in the MPLS technique for generating equations, decreasing the effect of irrelevant large spectroscopic variations. Absorption levels are generally altered due to the variation in the scattering of light and path length variation caused by intervention in sample particles and light. Linear calibration and spectral interpretation of NIR spectra becomes very complex and difficult due to the alterations (13). Spectral pre-processing was employed to diminish the multiplicative effect of particles size and scattering including scatter correction and derivatization techniques (22). SNV works by removing the mean from each spectrum, followed by dividing the value of each signal by the SD of the entire spectrum to center it around zero. Along with SNV, detrend is another approach to correct behavior shift. SNV with detrend (DT) in the present study was employed to avoid any noise in the NIRS signal baseline. Table 2 summarizes the calibration models by MPLS for protein, starch, TDF, phenols, and phytates in homogenized cowpea flour. For the development of calibration equations for various parameters, several mathematical treatments like “2,4,4,1”, “2,4,6,1”, “2,8,8,1”, and “3,4,4,1” were finalized. Our calibration equation was based on the highest 1-VR and RSQ_internal_, lowest SEC(V) values. Resolution of spectra can be improved by using derivatives 2 and 3, which eliminates baseline shifts and superimposed peaks. The signal-to-noise ratio in the spectral region caused due to erratic high-frequency perturbations can be improved by using gaps 4 and 8 and smoothening (S1, S2). Calibration equations were generated by removing a few outliers (<10), which occurred due to scanning or analytical errors and were removed. As given in the Table 1, RSQ_internal_ for different traits was obtained for protein (0.800), starch (0.997), TDF (0.934), phenols (0.719) and phytates (0.985) for the given mathematical treatments “2,4,6,1”, “2,8,8,1”, “2,4,4,1”, “3,4,4,1”, and “2,8,8,1” respectively.

**Table 2:** Calibration statistics of 81 cowpea accessions.

**Traits**	***N***	**Outliers**	**Range**	**Math treatment**	**Mean**	**RSQ**	**Slope**	**SD**	**SEC (V)**
Protein	81	4	20.0–27.8%	2,4,6,1	23.9	0.800	1.000	1.29	1.23
Starch	81	7	26.7–38.7%	2,8,8,1	32.7	0.997	1.004	2.00	0.063
TDF	81	5	12.2–22.4%	2,4,4,1	17.3	0.934	0.954	1.70	1.11
Phenols	81	4	0.08–0.545%	3,4,4,1	0.251	0.719	1.000	0.098	0.085
Phytic Acid	81	5	0.583–1.62%	2,8,8,1	1.10	0.985	0.997	0.173	0.266

N, number of samples; RSQ, coefficient of determination; SD, standard deviation; SEC(V), standard error of cross validation.

### Validation of the NIRS model

**Figure 3:**
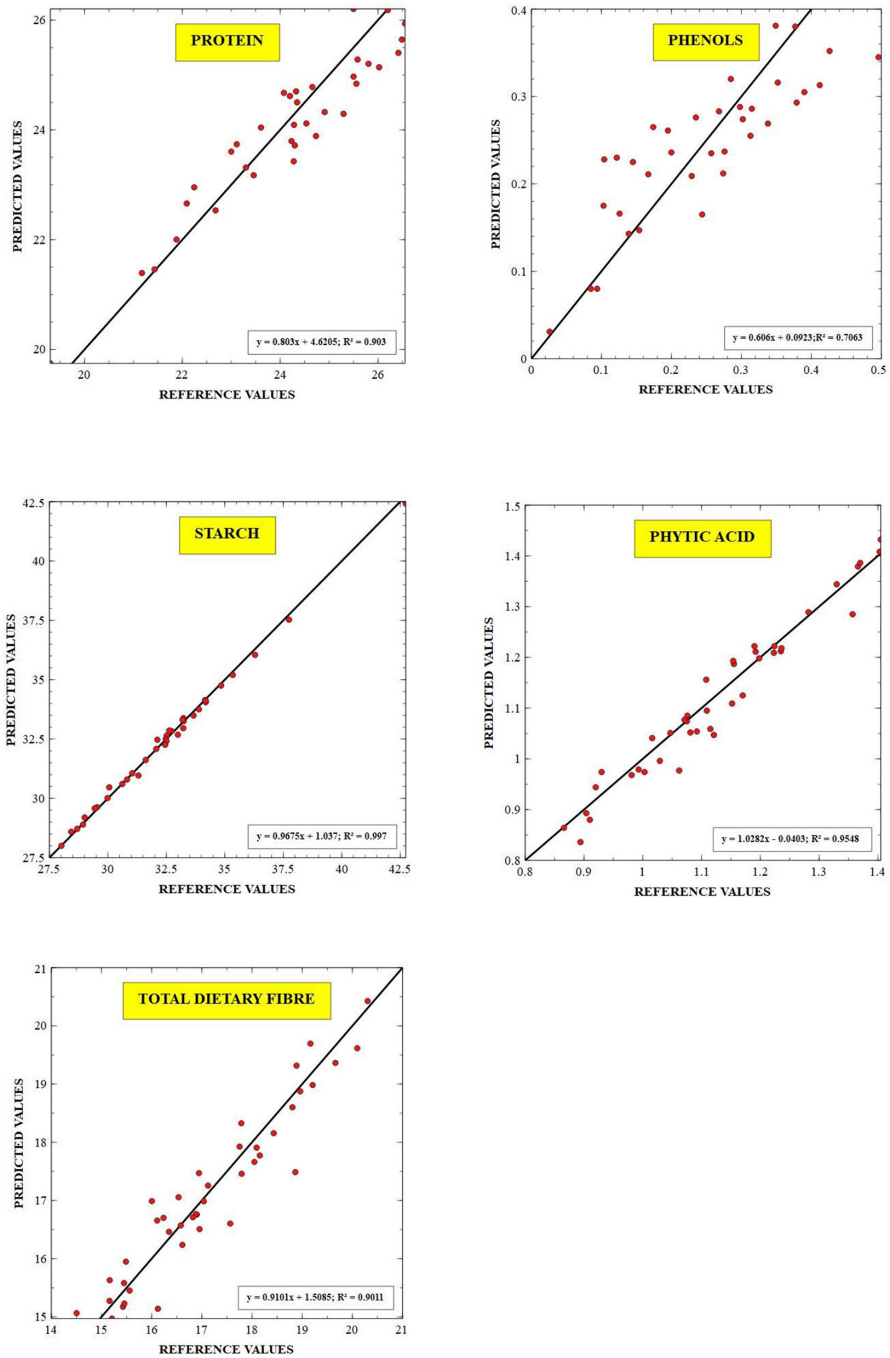
Scatter plot between the reference vs. predicted values for protein, starch, TDF, total dietary fiber, phenols, and phytic acid. RSQ_external_–coefficient of determination for validation.

The external validation statistics for the given traits, protein, starch, TDF, phenols, and phytates of 40 samples are shown in Table 3. No outliers were removed in external validation to show higher prediction power and ensure the robustness of developed models. The best fit models were chosen based on higher RSQ_external_, RPD, and low SEP, SD, slope, and bias values. To authenticate the model's validity, RPD value was used, which considers both SEP and variation in values and is more precise than SEP(C) (45). Towett et al. (14) found an RSQ_external_ of 0.93 for crude protein in cowpea leaves, whereas Pande and Mishra (18) found an RSQ_external_ of 0.97 for phytates in green gram seeds using FT-NIRS. The regression plot of predicted values versus reference values for protein, starch, TDF, phenol and phytic acid are described in Figure 3 respectively. These plots of predicted values versus reference values were developed through Win ISI III project manager V 1.50. Models for proteins, starch, TDF, phenols, and phytates displayed RPD values of 2.80, 5.32, 3.28, 1.78, and 4.45, respectively, denoting the model's excellent prediction power. Our RPD values for protein are in agreement with Ishikawa et al. (2.88) in cowpea (12). RPD value for phenolics (1.78) which shows that the model can distinguish between higher and lower values. Slope denotes the change in predicted values with a unit change in reference values. The ideal value of the slope is 1, and any value close to 1 would indicate an accurate model. The values of slope in our study for different traits were protein (1.12), starch (1.03), TDF (0.954), phenols (1.18), and phytates (0.929). In determining the model accuracy, bias is an important indicator of similarity between reference and predicted values of the model (23). When the reference and predicted values are the same, the bias would be equal to zero, which is the ideal value for bias. An underestimating model will be signified by negative bias, and overestimating model will be signified by positive bias (46). The values of bias for different traits were 0.197 (protein), 0.029 (starch), −0.026 (TDF), phenols 0.003 (phenols), and 0.009 (phytates), where all the developed models were found to be overestimating except TDF which is found to be underestimating.

**Table 3:** External validation statistics of 40 cowpea accessions.

**Traits**	***N***	**Range**	**Math treatment**	**Mean**	**RSQ**	**Slope**	**Bias**	**SD**	**SEP**	**RPD**
Protein	40	19.3–26.5%	2,4,6,1	24.2	0.903	1.122	0.197	1.67	0.598	2.80
Starch	40	28.1–42.7%	2,8,8,1	32.4	0.997	1.028	0.029	2.81	0.528	5.32
TDF	40	14.5–20.3%	2,4,4,1	17.2	0.901	0.954	−0.026	1.49	0.454	3.28
Phenols	40	0.03–0.496%	3,4,4,1	0.247	0.706	1.179	0.003	0.114	0.064	1.78
Phytic acid	40	0.866–1.40%	2,8,8,1	1.12	0.955	0.929	0.009	0.147	0.033	4.45

N, number of samples; RSQ, coefficient of determination; SD, standard deviation; SEP, standard error of performance; RPD, ratio of performance to deviation.

To determine whether the mean of a dependent variable is the same as the analytical and predicted values for the examined biochemical parameters, a paired *t*-test with a 95% confidence interval was performed. In our study, the *p*-value came out more than 0.05, indicating the accuracy and reliability of the models (Table 4). The *p*-values of protein, starch, TDF, phenols, and phytates are 0.158, 0.512, 0.637, 0.639, and 0.114 respectively. Hence no statistically significant differences came out between the means in the NIRS method and standard methods used to analyze the traits.

**Table 4:** Paired sample t-test at 95% confidence interval.

**Pairs**	**Paired differences**							
	**Mean**	**SD**	**SEM**	**95% confidence interval of the difference**		***t*-value**	**DF**	***p*-value**
				**Lower**	**Upper**			
Protein reference—protein predicted	0.135	0.260	0.0934	−0.0549	0.325	1.44	40	0.158
Starch reference—starch predicted	0.0179	0.02	0.0270	−0.0368	0.0725	0.662	40	0.512
TDF reference—TDF predicted	0.0350	0.06	0.0736	−0.114	0.184	0.475	40	0.637
Phenol reference—phenol predicted	0.00506	0.0318	0.0107	−0.0167	0.0268	0.473	40	0.639
Phytate reference—phytate predicted	0.00850	−0.008	0.00526	−0.00214	0.0191	1.62	40	0.114

SD, standard deviation; SEM, standard error of mean; DF, degree of freedom.

### Applicability of the developed models

The validated models were used to predict a sample set of 202 cowpea accessions for use in screening germplasm resources. To ensure the reliability of developed equations, 10% of samples were analyzed by standard methods. The predicted data were arranged in ascending order, and every 11th sample was selected for wet chemistry analysis. The result of predicted and laboratory values are given in Table 5. Correlation studies between the datasets show high correlation for protein (*r* = 0.97, *p* < 0.001), starch (*r* = 0.93, *p* < 0.001), TDF (*r* = 0.95, *p* < 0.001) while slightly low correlation was observed for phenols (*r* = 0.55, *p* < 0.01) and phytic acid (*r* = 0.77, *p* < 0.01) (Table 6). Strict parallel analysis is used to determine the difference in the means and standard deviations of two datasets where in our study there was agreement in the consistency of results, showing high reliability scores (unbiased) for protein (0.91), 0.92 (starch), 0.97 (TDF), 0.87 (phytic acid) and comparatively low reliability for phenols (0.64) (Table 6).

**Table 5:** Prediction and wet chemical values of selected cowpea accessions.

**Acc no**.	**%TDF**		**%Phytates**		**%Phenol**		**%Protein**		**%Starch**
	**Pred**	**Val**	**Pred**	**Val**	**Pred**	**Val**	**Pred**	**Val**	**Pred**	**Val**
NIC23093	21.0	20.6	1.51	1.54	0.251	0.354	24.3	25.3	32.0	30.9
IC52099	21.9	22.2	1.28	1.31	0.215	0.345	23.7	24.6	30.9	31.1
IC140239	24.6	25.1	1.60	1.63	0.157	0.134	21.4	21.7	36.7	40.1
IC209139	20.4	21.3	1.33	1.26	0.204	0.311	24.1	25.0	32.4	35.7
EC240841	17.8	18.8	1.34	1.45	0.288	0.263	24.0	24.6	31.6	32.4
EC241015	20.9	21.0	1.44	1.34	0.228	0.211	23.4	24.1	32.3	33.4
IC257430	18.7	19.4	1.27	1.22	0.259	0.288	24.4	25.3	31.2	32.1
IC265570	19.6	18.5	1.44	1.40	0.274	0.3	25.6	26.1	30.0	30.1
IC326996	18.4	18.7	1.46	1.62	0.24	0.23	23.4	24.2	34.1	33.9
IC341244	21.0	21.7	1.55	1.88	0.246	0.248	22.7	23.5	34.0	34.1
IC372718	19.2	19.2	1.35	1.23	0.262	0.258	23.9	24.6	30.5	31.2
IC397907	18.2	18.7	1.46	1.34	0.276	0.287	26.0	27.3	30.1	30.1
IC426824	17.5	18.1	1.47	1.45	0.297	0.32	23.1	24.2	33.5	33.6
IC488259	19.9	20.0	1.33	1.39	0.282	0.313	24.9	25.3	29.7	30.0
IC546253	20.0	20.1	1.49	1.45	0.275	0.266	23.5	24.4	32.9	33.2
EC724421	20.2	20.2	1.59	1.62	0.24	0.288	24.4	25.5	31.9	31.7
IC91521A	20.9	21.5	1.53	1.56	0.284	0.301	24.6	25.6	31.1	31.6
EC240917	21.4	22.5	1.75	1.66	0.20	0.244	22.8	23.5	35.2	36.9
IC724382	21.5	22.6	1.45	1.40	0.194	0.228	21.5	21	35.7	39.0
% MEAN	20.2	20.5	1.5	1.5	0.2	0.3	23.8	24.5	32.4	33.2
STDEV	1.64	1.75	0.12	0.17	0.04	0.05	1.14	1.40	1.95	2.82

ACC, accession; TDF, total dietary fiber; PRED, predicted values; VAL, validated values; STDEV, standard deviation.

**Table 6:** Reliability analysis between predicted and laboratory validated values using strict parallel method.

		**MEANS**	**STDEV**			
**Trait**	***N***	**Pred**	**Lab val**	**Pred**	**Lab val**	**Reliability of scale**	**Correlation (pred/lab val)**
Protein	19	23.7	24.5	1.18	1.43	0.91	0.97***
Starch	19	32.4	33.2	2.00	2.91	0.92	0.93***
TDF	19	20.1	20.5	1.69	1.79	0.97	0.95***
Phenols	19	0.24	0.27	0.037	0.051	0.64	0.55**
Phytic acid	19	1.45	1.46	0.121	0.171	0.87	0.77***

N, number of samples; PRED, predicted values; LAB VAL, laboratory validation; STDEV, standard deviation; ***p < 0.001; **p < 0.01

## Conclusion

In our study for the rapid prediction of protein, starch, TDF, phenols, and phytates, NIRS was found to be a potential tool. The present work is the first report on the development of prediction models of protein, starch, TDF, phenols, and phytates in cowpea through the MPLS regression method based on the NIR spectroscopy method. MPLS regression was used to develop models which have shown suitability for all the traits. Good RSQ_external_ and RPD values have been found for multi-trait parameters. Model applicability studies confirmed that these models could predict the traits of diverse cowpea germplasm with excellent accuracy and precision. The use of NIR models substantially reduced the cost and time for analyzing multiple traits in cowpea germplasm without compromising on data quality. Thus, these developed models will facilitate high throughput screening of large cowpea germplasm present in the national and international gene banks throughout the world, for identifying traits specific germplasm and selecting desirable chemotypes in any genetic background with huge application in cowpea crop improvement program across the world. However, these prediction models have been developed in the flour of cowpea but prediction models should also be developed in grain, as it will facilitate the screening of cowpea germplasm in a completely non-destructive way.

## Data availability statement

The original contributions presented in the study are included in the article/supplementary material, further inquiries can be directed to the corresponding author/s.

## Author contributions

SRP and RJ: biochemical profiling, NIRS modeling, and preparing draft manuscript. AB: reference data generation. KT: provided agronomically diverse cowpea accessions. KG, DW, GM, and JCR: review and critical evaluation of manuscript. SK: statistical analysis and interpretation. AR: revision and resources. RB: conceptualization, planning, and supervision of study. All authors contributed to the article and approved the submitted version.

## Funding

This study was supported by funding from Department of Biotechnology, Government of India under project on minor pulses (No. BT/Ag/Network/Pulse-I /2017-18, date:187 24th Oct 2018). In-house project on Biochemical evaluation of Field and vegetable crops. DBT funded and an international collaborative project Consumption of Resilient Orphan Crops & Products for Healthier Diets (CROPS4HD) which is co-funded by the Swiss Agency for Development and Cooperation, Global Programme Food Security (SDC GPFS) and executed in India through FiBL (Research Institute of Organic Agriculture), and Alliance of Bioversity and CIAT with ICAR-NBPGR as a lead partner. Junior Research Fellowship granted to SP by ICAR Research Grant No. 2(9)/2018-HRD dated 30th October 2018.

## Conflict of interest

The authors declare that the research was conducted in the absence of any commercial or financial relationships that could be construed as a potential conflict of interest.

## Publisher's note

All claims expressed in this article are solely those of the authors and do not necessarily represent those of their affiliated organizations, or those of the publisher, the editors and the reviewers. Any product that may be evaluated in this article, or claim that may be made by its manufacturer, is not guaranteed or endorsed by the publisher.

## Footnotes

^1^ http://fdc.nal.usda.gov

## Abbreviations

NIRS: near infrared reflectance spectroscopy
MPLS: modified partial least square
TDF: total dietary fiber
RSQ: coefficient of determination
RPD: ratio of performance to deviation
SEP: standard error of performance.
